# Thermostable Alkaline Phytase from *Alcaligenes* sp. in Improving Bioavailability of Phosphorus in Animal Feed: *In Vitro* Analysis

**DOI:** 10.5402/2013/394305

**Published:** 2013-12-13

**Authors:** Ponnuswamy Vijayaraghavan, R. Raja Primiya, Samuel Gnana Prakash Vincent

**Affiliations:** ^1^Centre for Marine Science and Technology, Manonmaniam Sundaranar University, Rajakkamangalam, Kanyakumari, Tamil Nadu 629 502, India; ^2^P. G. Department of Microbiology, Ayya Nadar Janaki Ammal College, Sivakasi, Tamil Nadu 626 124, India; ^3^International Centre for Nanobiotechnology, Centre for Marine Science and Technology, Manonmaniam Sundaranar University, Rajakkamangalam, Kanyakumari District, Tamil Nadu 629 502, India

## Abstract

A bacterial isolate, *Alcaligenes* sp. secreting phytase (EC 3.1.3.8), was isolated and characterized. The optimum conditions for the production of phytase included a fermentation period of 96 h, pH 8.0, and the addition of 1% (w/v) maltose and 1% (w/v) beef extract to the culture medium. This enzyme was purified to homogeneity and had an apparent molecular mass of 41 kDa. The optimum pH range and temperature for the activity of phytase were found to be 7.0-8.0 and 60°C, respectively. This enzyme was strongly inhibited by 0.005 M of Mn^2+^, Mg^2+^, and Zn^2+^. *In vitro* studies revealed that the phytase from *Alcaligenes* sp. released inorganic phosphate from plant phytates. Phytase released 1930 ± 28, 1740 ± 13, 1050 ± 31, 845 ± 7, 1935 ± 32, and 1655 ± 21 mg inorganic phosphate/kg plant phytates, namely, chick pea, corn, green pea, groundnut, pearl pea, and chick feed, respectively.

## 1. Introduction

Phytate-degrading enzyme (phytase, EC 3.1.3.8) preparations have a wide range of applications in animal and human nutrition. Besides that, these enzymes have also attracted considerable attention from both scientists and entrepreneurs in the areas of environmental protection and biotechnology. Phytases are of great interest in biotechnological applications, in particular for the reduction of phytate content in feed and food [1]. Phytases are capable of initiating the stepwise release of myoinositol and phosphoric acid, leading to the formation of myoinositol phosphate intermediates from phytate [2]. Most plant-origin foods have from 50% to 80% of their total phosphorus as phytate [3]. Phytate chelates essential minerals, binds to amino acids and proteins, inhibits digestive enzymes, and decreases the nutritive value of food [4]. Monogastric animals poorly utilized phytate-bound phosphorus, due to insufficient phytate-degrading activity in the gut [5]. Therefore, hydrolysis of phytate is desirable for releasing valuable nutrients for beneficial utilization. The addition of phytate-degrading enzymes can improve the nutritional value of plant-based foods by enhancing protein digestibility and mineral availability through phytate hydrolysis during digestion in the stomach or during food processing [6], thus reducing the phosphorus excretion of animals [7]. 

Only a limited number of bacterial phytases have been reported and studied [8]. Phytase has been isolated from bacteria such as *Escherichia coli *[9], *Pseudomonas *sp. [10], anaerobic rumen bacteria, particularly *Selemonas ruminantium, Megasphaera elsdenii, Prevotella *sp., *Mitsuokella multiacidus *[11], and *Raoultella *sp. [12]. Numerous studies have shown the effectiveness of supplemental microbial phytases in improving the utilization of phosphate from phytate [13]. Naturally occurring phytases having the required level of thermostability for application in animal feeds have not been found [14]. Hence, the main objective of the present study was to characterize alkaline- thermostable phytase from *Alcaligenes *sp., and possible application in *in vitro* digestion of plant phytate was evaluated. A study of this kind will improve our knowledge on the biotechnological application of phytase in feed industry.

## 2. Materials and Methods

### 2.1. Bacterial Culture and Screening

The bacterial isolate, *Alcaligenes *sp., was obtained from the Microbiology Laboratory, Centre for Marine Science and Technology, M. S. University, Rajakkamangalam, Tamil Nadu, India. To screen phytase-producing microorganism effectively, the plate agar method was followed [15]. Sodium phytate (2%, w/v) was added as substrate in plate screening media. The *Alcaligenes *sp. was grown on this solid medium and incubated at 37°C for 48 h. Phytase plate screening was carried out by washing the colonies with distilled water from the agar surface and flooding the plate with 2% (w/v) cobalt chloride solution and incubated for 30 min at room temperature. Then replaced the cobalt chloride solution with a freshly prepared solution containing equal volume of 6.25% (w/v) ammonium molybdate and 0.42% (w/v) ammonium vanadate solution. Following further 30 min incubation the solution removed and the plates were examined for zones of clearing which indicates phytase activity.

### 2.2. Phytase Production

Phytase-secreting *Alcaligenes *sp. was subjected for phytase production in the production medium (containing 1 litre: sodium phytate, 10 g; (NH_4_)_2_SO_4_, 1 g; MgSO_4_·7H_2_O, 0.1 g; CaCl_2_·2H_2_O, 0.1 g; trace element solution, 1.0 mL; KCl, 0.7 g; glucose, 1 g; D-mannose, 1 g). The pH of the medium was adjusted to 7.0 by using 1N HCl/1N NaOH. Sodium phytate was filter-sterilized separately, and added after autoclaving, to the production medium. A loopfull of bacterial culture was transferred to 150 mL of phytase production medium in a 250-mL Erlenmeyer flask, and the culture was incubated at 37°C with shaking at 150 rpm. For the determination of the growth curve, 5 mL of the culture medium was withdrawn at regular intervals of 12 h and the cell density determined at 600 nm up to 120 h. To study the optimum incubation time for phytase production, the culture was withdrawn every 12 h and centrifuged at 10,000 ×g for 15 min. The culture supernatant was assayed for determining enzyme activity. Effect of pH on enzyme production was studied by adjusting the culture medium pH to 6.0, 7.0, 8.0, 9.0, and 10.0 by the addition of 1 N HCl/NaOH prior to sterilization. To study the effect of carbon and nitrogen sources on enzyme production, the organism was grown in the production medium containing additional 1% (w/v) carbon (arabinose, dextrose, maltose, sucrose, trehalose, mannose, and starch) and nitrogen sources (urea, ammonium sulphate, peptone, ammonium chloride, and beef extract). All experiments were conducted in triplicates and average values were reported.

### 2.3. Crude Enzyme Preparation and Phytase Assay

Bacterial culture was prepared for assay as suggested by Yanke et al. [11]. A cell-free extract was prepared from 96h-old culture by centrifuging it at 10,000 ×g for 15 min. The culture supernatant was assayed for determining phytase activity, which was based on the estimation of inorganic phosphate released on hydrolysis of phytic acid, at 37°C. One unit of enzyme activity was defined as the amount of enzyme that liberates 1 *μ*mol of inorganic phosphate per minute under standard assay conditions. The liberated inorganic phosphate was measured according to the ammonium molybdate method [16]. The total protein content of the sample was estimated as described by Lowry et al. [17], and bovine serum albumin was used as the standard.

### 2.4. Properties of *Alcaligenes* Phytase

The optimum pH for the enzyme activity was determined by using the following buffers (0.1 M): citrate buffer (pH 4.0), succinate buffer (pH 5.0-6.0), tris-acetate buffer (pH 7.0), tris-HCl buffer (pH 8.0), and glycine-NaOH buffer (pH 9.0). The stability of the enzyme at various pH was examined by incubating the enzyme solution in buffers ranging in pH from 4 to 9 at 37°C for 30 min; enzyme activity was assayed as described earlier. The temperature profile of the enzyme was determined by performing the routine enzyme assay at varying incubation temperatures (30, 40, 50, 60, and 70°C). To determine the thermal stability of the enzyme, it was incubated for 30–70°C for 30 min before performing the routine enzyme assay. The effect of divalent ions and chemicals on enzyme activity was determined by incubating the enzyme with various divalent ions and chemicals (0.005 M), namely, Ca^2+^, Mn^2+^, Zn^2+^, Cu^2+^, Mg^2+^, ethylenediaminetetraacetic acid (EDTA), dithiothreitol, and *β*-mercaptoethanol for 30 min. The enzyme activity was assayed as described earlier. 

### 2.5. Purification of Extracellular *Alcaligenes* Phytase

The crude enzyme preparation (140 mL) was fractionated with ammonium sulphate (60% to 80% saturation) as suggested by Fu et al. [18]. The protein precipitate was dissolved in a minimal volume of double-distilled water and the resulting enzyme was dialyzed against the buffer A (tris-HCl buffer, 0.025 M, pH 8.0) overnight at 4°C. The dialyzed sample was subjected to Diethyl-aminoethyl cellulose (DEAE cellulose) chromatography (Merck, Bangalore, India) using buffer A, and the bounded protein was eluted with a linear gradient of NaCl (0-1 M). The fractions having phytase activity were combined and concentrated using ammonium sulphate (80% saturation) and dialyzed against buffer B (tris-HCl buffer, 0.05 M, pH 8.0). The concentrated sample was loaded on a preequilibrated sephadex G-75 column (Amersham Biosciences, SE-751 84, Uppsala, Sweden) and eluted with buffer B. 

### 2.6. Polyacrylamide Gel Electrophoresis

Sodium dodecyl sulphate-polyacrylamide gel electrophoresis (SDS-PAGE) was performed according to Laemmli [19]. The stacking gel was 5% (w/v) polyacrylamide, and the separating gel was 11% (w/v) polyacrylamide. The active fraction obtained from the sephadex G-75 column was subjected to electrophoresis. After electrophoresis, the gel was treated and stained for phytase activity as described by Yanke et al. [11], with a cobalt chloride solution followed by an ammonium molybdate/ammonium vanadate solution. The gel was documented using a gel documentation system (Syngene, Cambridge, CB4 1TF, UK) and the molecular weight of the purified enzyme determined.

### 2.7. *In Vitro* Hydrolysis of Plant Phytate

An *in vitro *experiment was performed to study the hydrolysing efficacy of *Alcaligenes *phytase on plant phytate [18]. Briefly, plant phytates from various sources, namely, chick pea, corn, green pea, groundnut, pearl pea, and chick feed named “Layer Mash” brought from the local market were used. All materials were ground into coarse powder and sieved (0.2-0.3 mm). Two grams of the above material were placed in a 50-mL conical flask. To this, 10 U crude enzyme and 5 mL of buffer B were added and the mixture was placed in an orbital shaker at 150 rpm for 30 min. A control experiment was carried out separately; for this, 10 mL of buffer B alone was applied to the raw materials. To the reaction mixture, 10 mL trichloroacetic acid (15%) was added to stop the reaction. The released inorganic phosphorus was determined [19]. 

## 3. Results and Discussion

### 3.1. Screening of Phytase Secreting *Alcaligenes* sp

The isolate, *Alcaligenes *sp. secreted alkaline phytase when it was grown on phytase screening medium. This organism produced a 2.0-mm zone around the colony after 48 h. This organism was used for further studies. 

### 3.2. Growth and Phytase Production

**Figure 1:**
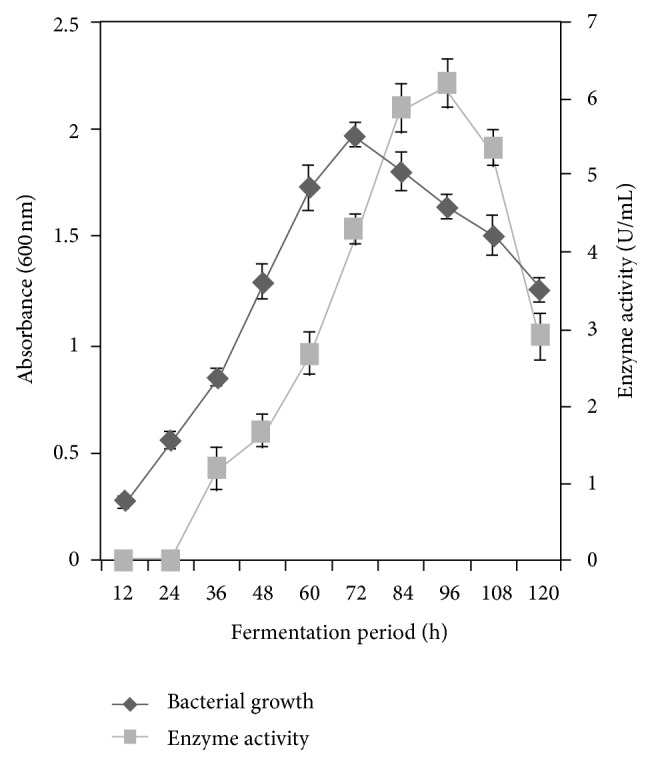
Growth and production of phytase from *Alcaligenes *sp. The isolate was inoculated and incubated for 120 h at 37°C. The culture was withdrawn every 12 h, and the growth enzyme activity was determined. The result was the mean of three different repeats. The error bar indicates standard deviation.

The *Alcaligenes *sp. attained maximum growth after 72 h of fermentation, and the absorbance was 1.972 ± 0.036 at 600 nm. The absorbance declined to 1.728 ± 0.027, 1.615 ± 0.039, 1.619 ± 0.781, and 1.407 ± 0.033 at 84, 96, and 108 h of incubation, respectively. The isolate produced an interesting phytase when grown in minimal medium containing sodium phytate as the sole source of phosphorus. The enzyme secretion increased to its maximum of 6.202 ± 0.082 U/mL at 96 h of fermentation. Enzyme production was found to be 0.008 ± 0.005, 1.811 ± 0.221, 4.351 ± 0.192, and 3.610 ± 0.191 U/mL at 24, 48, 72, and 120 h of fermentation, respectively, (Figure 1). In this organism, phytase was expressed on the onset of late log phase. The reduction in enzyme yield after the optimum period was probably due to the depletion of nutrients that are available to microorganism. This result was in accordance with the observation made with *Yersinia kristensenii *[18].

### 3.3. Effect of pH on Enzyme Production

Effect of pH on enzyme production was studied by culturing the organism at various pH levels (6.0–10.0) using 1 N HCl/NaOH. Phytase production was 1.5 ± 0.15, 2.96 ± 0.16, 2.0 ± 0.23, 1.7 ± 0.10 U/mL at pH 6.0, 7.0, 9.0, and 10.0, respectively. Enzyme production was found to be high at pH 8.0 (4.9 ± 0.18 U/mL). At high pH level (9.0), phytase production decreased. At higher pH, the metabolic action of the bacterium may be suppressed and thus enzyme production decreased. This result was in accordance with the observations made with *Mitsuokella jalaludinii *by Lan et al. [20].

### 3.4. Effect of Carbon and Nitrogen Sources on Enzyme Production

Phytase production was found to be high (6.2 ± 0.018 U/mL) in the production medium supplemented with 1% (w/v) maltose. Enzyme production was 5.0 ± 0.032, 4.33 ± 0.01, 4.8 ± 0.05, 3.47 ± 0.09, and 3.07 ± 0.008 U/mL in the production medium containing starch, dextrose, trehalose, sucrose, and arabinose, respectively. Mannose (1%, w/v) repressed the production of phytase. Similar results were obtained with *Bacillus *sp. KHU-10 [21]. Phytase production was high (6.5 ± 0.026 U/mL) in the production medium containing beef extract (1%, w/v) as the sole source of nitrogen. Enzyme production was 3.35 ± 0.05, 3.0 ± 0.03, 4.6 ± 0.13, and 5.2 ± 0.08 U/mL in production medium containing urea, ammonium sulphate, peptone, and ammonium chloride, respectively. This result was in accordance with the observation made with *Bacillus *sp. KHU-10 [21]. 

### 3.5. Effect of pH on Enzyme Activity and Stability

**Figure 2:**
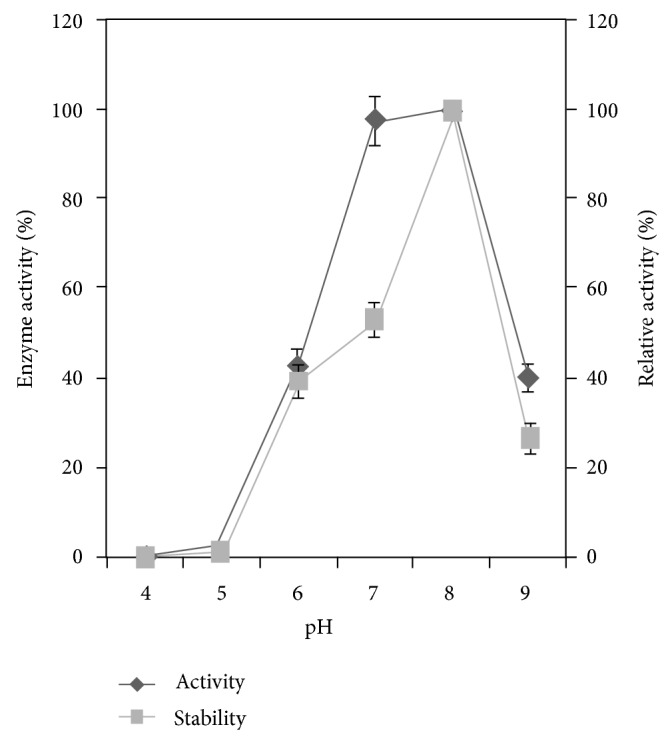
Effect of pH on phytase activity and stability. The *Alcaligenes *sp. was inoculated in the minimal medium containing 1% (w/v) sodium phytate as the sole phosphorus source and incubated at 37°C for 144 h in an orbital shaker at 150 rpm. The result was the mean of three different repeats. *Error bar *standard deviation.

In *Alcaligenes* sp. phytase activity was found to be high (100% activity) at pH 8.0. The relative enzyme activity was 0.9  ±  0.13%, 3.4  ±  0.18%, 44.5  ±  3.4, 98  ±  5.5%, and 40  ±  2.2% at pH 4.0, 5.0, 6.0, 7.0, and 9.0, respectively. This enzyme was also highly stable at pH 8.0 at which the relative enzyme activity was 100%. The relative enzyme activity was 0.4  ±  0.16, 40  ±  0.21, 47  ±  0.18, and 27  ±  0.24%, at pH 4.0, 5.0, 6.0, 7.0, and 9.0, respectively, (Figure 2). This property was in accordance with the previously reported results. Some bacterial phytases have broad pH optima but are shifted towards a more basic pH range [22, 23]. This enzyme was highly stable at pH 8.0 (100% relative activity). 

### 3.6. Effect of Temperature on Enzyme Activity and Stability

**Figure 3:**
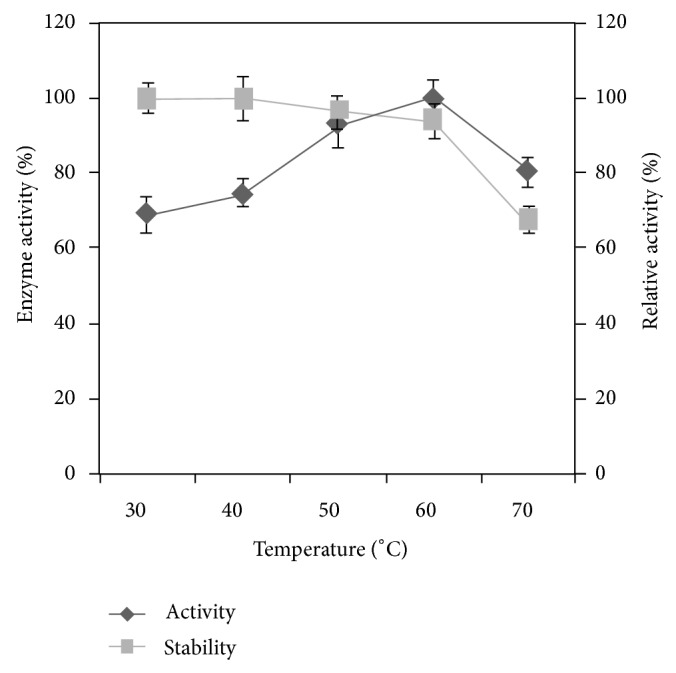
Effect of temperature on enzyme activity and stability. The enzyme sample was incubated with substrate for 30 min at temperatures ranging from 30 to 70°C. The result was the mean of three different repeats. *Error bar *standard deviation.

The effect of phytase activity was studied by incubating the reaction mixture at various temperatures (30–70°C). The *Alcaligenes *phytase was highly active (100% activity) at 60°C (Figure 3). Optimal temperatures in which most phytases are active vary from 37 to 77°C [24]. Similar result was reported by Dvořáková [4] with *Bacillus subtilis *and with *Pseudomonas syringae *[25]. The relative enzyme activity was found to be high (100%) at 60°. The relative enzyme activity was 59  ±  1.6%, 68  ±  2.7%, 92  ±  3.4%, and 86  ±  5.3% at 30, 40, 50, and 70°, respectively. Results indicated that the enzyme was stable up to 60 degrees of denaturing temperature. The relative enzyme activity was 64.7  ±  4% at 70°C (Figure 3). This result shows that *Alcaligenes *phytase is relatively thermo-stable and is preferable for use in feed industry. This enzyme activity was comparable with *Bacillus laevolacticus *phytase [26].

### 3.7. Effect of Ions and Chemicals on Enzyme Activity

The effect of divalent ions on enzyme activity was investigated by allowing the divalent ions (0.005 M) to react with the phytase sample. Divalent ions have been found to modulate *Alcaligenes *sp. phytase activity. However, it is difficult to determine whether the inhibitory effect of various metals is due to the direct binding of the metal ions to the enzyme, or whether the metal ions form poorly soluble complexes with phytic acid, thereby decreasing the active substrate concentration. Among divalent, Ca^2+^ enhanced the relative enzyme activity (103  ±  2.89%) when compared with the control (100%). Mn^2+^, Zn^2+^, Cu^2+^, and Mg^2+^ inhibited the enzyme activity and the relative activity was 46  ±  3.8%, 39  ±  2.92%, 73  ±  1.3%, and 21  ±  0.8%, respectively. EDTA also inhibited phytase activity (49  ±  1.3%). Similar results were obtained with *Enterobacter *sp. [23] and *Yersinia kristeensenii *[18]. 

### 3.8. Purification of *Alcaligenes* Phytase

**Figure 4:**
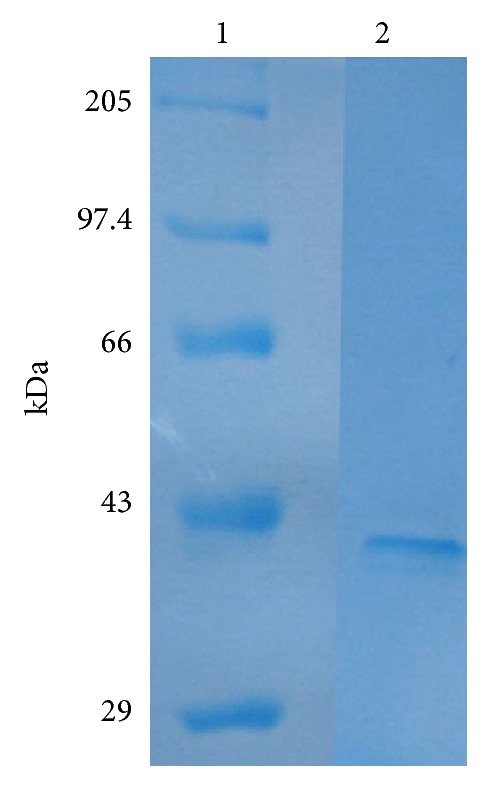
Sodium dodecyl sulphate polyacrylamide gel electrophoresis (11%) of the purified phytase from* Alcaligenes *sp. Lane 1: molecular mass standards; Lane 2: purified sample from sephadex G-75.

Phytase was purified to homogeneity by sequential ammonium sulphate precipitation, anionic exchange chromatography, and gel filtration chromatography. The DEAE cellulose chromatography was still the major technique for purification of *Alcaligenes *phytase, because majority of contaminating proteins were removed at this step. In this step 3.57-fold purification was achieved with 25.3% yield. The recovery and purification were 7.07%- and 4.75-fold, respectively, after sephadex G-75 gel filtration chromatography. The purification procedure of phytase is summarized in Table 1. The molecular weight of the enzyme was found to be 41 kDa (Figure 4). A two-step chromatographic process, similar to that employed with *Aspergillus ficuum* [27], was followed. Homogeneity of the purified enzyme was revealed by SDS-PAGE which showed a single band with an apparent molecular mass of 41 kDa (Figure 4). This result similar with a molecular mass was reported in bacteria [28]. 

### 3.9. Invitro Hydrolysis of Plant Phytate

In the present study various sources of plant phytates were used to investigate the effect of phytate, and the released inorganic phosphorus was measured. Plant phytates, namely, chick pea, corn, green pea, groundnut, pearl pea, and chick feed named “Layer Mash” were chosen as the substrate for *in vitro *hydrolysis. The maximal inorganic phosphorus release was obtained with pearl pea. The released inorganic phosphorus from these sources was significantly high (Table 2) than other reported results. A similar finding was reported by Fu et al. [18] with *Yersinia kristeensenii.* This property of the *Alcaligenes *phytase makes this a potential supplement in the animal feed industry. The plant phytate sources mentioned above are widely used as raw materials in the animal feed industry. Phytase cannot be fully used by agastric fish like carps, in which the pH of the digestive tract is about 6.5–8.4. Interestingly, *Alcaligenes *phytase was active and stable at this range of pH. Thus, this phytase could be used effectively in the feed of monogastric animals and other organisms. 

## 4. Conclusions

Phytate-rich plant feed restricts the bioavailability of phosphorus along with other minerals. It is clear that supplemental phytase can enhance the bioavailability of phosphorus and other minerals in plant-based feed. The *Alcaligenes *phytase had an optimum pH at alkaline range (7.0-8.0) and, fortunately, the fish gut pH fell at this range. Hence, this phytase could be used effectively to increase the bioavailability of phosphorus in feed. Further investigation about phytase application in fish feed is needed to study in *in vivo.* The progress in biotechnology of phytase is remarkable and attracting worldwide attention. Overproduction of phytase can be achieved by physical, chemical methods of mutagenesis and through recombinant DNA technology.

## Figures and Tables

**Table 1:** Summary of the purification of the phytase from *Alcaligenes *sp.

Purification step	Total activity (U)	Total protein (mg)	Specific activity (U/mg)	Purification (fold)	Yield (%)
Crude enzyme	980	182	5.38	1.0	100
80% (NH_4_)_2_SO_4_	562	84.6	6.64	1.2	57.3
DEAE cellulose	248	12.9	19.2	3.57	25.3
Sephadex G-75	69.3	2.8	25.7	4.75	7.07

**Table 2:** *In vitro* hydrolysis of plant phytate by alkaline-stable phytase from *Alcaligenes *sp.

Phytate source	Released inorganic phosphorus (mg/kg raw material)
Chick pea	1930 ± 23
Corn	1740 ± 86
Green pea	1050 ± 61
Ground nut	845 ± 35
Pearl pea	1935 ± 42
Chick feed	1655 ± 25
